# Advancements in Pharmacological Treatment of Alzheimer’s Disease: The Advent of Disease-Modifying Therapies (DMTs)

**DOI:** 10.3390/brainsci14100990

**Published:** 2024-09-29

**Authors:** Qiong Wang, Sihui Chen, Junhui Wang, Huifang Shang, Xueping Chen

**Affiliations:** 1 Department of Critical Care Medicine, West China Hospital, Sichuan University, Chengdu 610041, China; 13709068150@163.com; 2 Department of Neurology, West China Hospital, Sichuan University, Chengdu 610041, China; chensihuiyyds@163.com (S.C.); hfshang2002@126.com (H.S.); 3 Lunenfeld-Tanenbaum Research Institute, Mount Sinai Hospital, Toronto, ON M5G 1X5, Canada; justin.wangjunhui@gmail.com; 4 Thyropathy Hospital, Sunsimiao Hospital, Beijing University of Chinese Medicine, Tongchuan 727000, China

**Keywords:** Alzheimer’s disease, disease-modifying therapies (DMTs), monoclonal antibodies, therapy

## Abstract

The landscape of pharmacological treatment for Alzheimer’s disease (AD) has undergone significant transformations with the advent of disease-modifying therapies (DMTs) targeting β-Amyloid (Aβ) accumulation, one of the hallmark pathologies of AD. The approval and market introduction of monoclonal antibodies mark the dawn of a new era in AD therapeutics as well. Furthermore, considerable progress has also been made in the development of new drugs targeting non-Aβ and non-Tau protein pathways. These advancements are key in tackling the root causes of AD, offering hope for treatments that both relieve symptoms and slow disease progression, improving patient outcomes and quality of life. This review aims to provide a comprehensive update on the advances in drug development and application for AD, including those currently in clinical trials and those already approved for the market to treat patients.

## 1. Introduction

**Figure 1:**
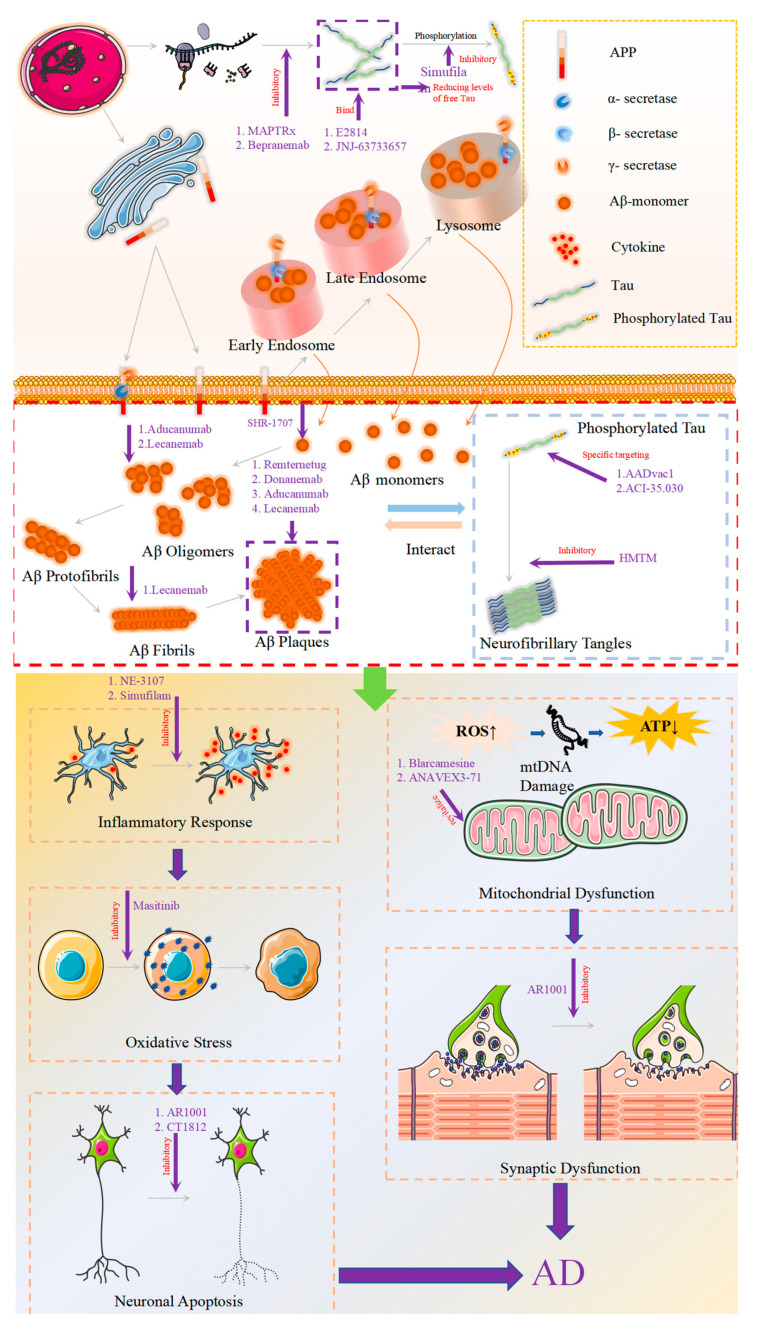
Pathogenic mechanisms of Alzheimer’s disease and drug targets, including pathways such as Aβ, Tau, synaptic dysfunction, neuroinflammation, oxidative stress, and mitochondrial dysfunction. Aβ stands for amyloid-beta.

Alzheimer’s disease (AD) is characterized by a convoluted pathology mechanism [1,2]. Currently, the most used pharmacological interventions for AD fall into three primary categories: (1) cholinesterase inhibitors, which include drugs like Donepezil and Rivastigmine [3,4,5]; (2) N-methyl-D-aspartate (NMDA) receptor antagonists, with Memantine serving as a representative medication [6]; and (3) drugs targeting the brain–gut axis (gut microbiota) to mitigate neuroinflammatory mechanisms, such as Gastrodin [7,8,9]. However, these treatments neither halt nor reverse disease progression. In recent years, the development and introduction of disease-modifying therapies (DMTs), exemplified by monoclonal antibodies targeting β-amyloid (Aβ) proteins, have ushered in a new era of pharmacological treatment for AD, aiming to directly affect disease progression and fundamentally improve patient prognosis rather than just alleviate symptoms [10]. Moreover, progress in drug research and development targeting non-Aβ and non-Tau protein therapeutic strategies may potentially further alter the disease trajectory of AD [11,12]. The present review aims to provide a comprehensive analysis and summary on the development and application of novel drugs for AD, including those in clinical trial phases, as well as those that have received market approval (Table 1). Schematic diagram summarizing the mechanisms of action of these drugs are presented in Figure 1.

## 2. Novel Drugs Targeting Aβ

The amyloid cascade hypothesis postulates that the abnormal production of and deficits in Aβ clearing lead to AD, positing the idea that effective treatments for AD should aim to restore the homeostasis of Aβ in the brain [13,14,15]. There are at least four main strategies to reduce Aβ levels in the brain: (1) preventing or reducing the over-production of Aβ, which could primarily be achieved through targeting the proteolytic enzymes (β-secretase and γ-secretase) that mediate the processing of amyloid precursor protein (APP) [16,17]; (2) inhibiting or ameliorating the aggregation of Aβ, by which certain anti-aggregation compounds could prevent Aβ oligomerization or promote the clearance of aggregates; (3) enhancing Aβ clearance, which could be achieved by regulating neprilysin, a zinc-dependent metalloprotease, acting as a crucial Aβ-degrading enzyme in the brain [18,19]; (4) clearing existing amyloid deposits through immunotherapy (passive or active immunization), such as monoclonal antibodies (mAbs) [20,21]. To date, the fourth strategy has been used clinically to treat AD and AD-related mild cognitive impairment (MCI) patients and achieved partial success with the above-mentioned passive immunization via intravenously administering mAbs targeting Aβ. This therapeutic strategy is currently becoming one of the representative success stories of novel DMTs in the treatment of neurodegenerative disease. However, over the past few decades, the development of Aβ antibodies has encountered significant difficulties. Currently, eight Aβ antibodies are either undergoing or have completed phase III clinical trials, while four have been terminated during this phase [22,23,24,25]. The reasons for these failures may include the inability of the drugs to alter disease progression or improve cognitive function, design flaws in clinical trials that failed to effectively select appropriate patient populations, and adverse effects that hindered the observation of efficacy [26]. Here, we primarily introduce the Aβ antibody drugs that have received market approval, as well as those currently undergoing clinical research, to provide reliable treatment options for researchers and patients.

### 2.1. Approved Aβ Monoclonal Antibodies

#### 2.1.1. Aducanumab

Aducanumab is a human IgG1 monoclonal antibody that targets the amino acids 3–7 of the Aβ peptides, specifically clearing Aβ plaques [27]. On 7 June 2021, the United States Food and Drug Administration (FDA) approved Aducanumab for the treatment of AD, making it the first DMT candidate treatment approved for AD [28]. The result of a phase III study showed that the highest dose of Aducanumab could significantly slow clinical deterioration as measured by the Clinical Dementia Rating–Sum of Boxes (CDR-SB), with a reduction of 22% compared to the placebo group [28]. However, Aducanumab could bring about incidence rates of 35% and 36% of amyloid-related imaging abnormalities with edema (ARIA-E) and/or microhemorrhages (ARIA-H) on brain MRI scan detection during the treatment. One-fourth of participants with imaging abnormalities demonstrated clinical symptoms, especially those carrying the *APOE ε4* allele [29].

#### 2.1.2. Lecanemab

Lecanemab, a humanized mouse monoclonal antibody 158, antagonizes the mid-domain Aβ structures in amyloid protofibrils associated with the Swedish familial *APP* mutation [30]. On 27 September 2022, the results of CLARITY AD (NCT03887455), which included 1795 patients with early-stage AD, demonstrated that Lecanemab met its primary endpoint [30,31]. Lecanemab showed a statistically significant difference in CDR-SB scores among patients with or without treatment, indicating a clinical slowdown of disease progression. Participants received intravenous infusions of 10 mg/kg of Lecanemab or a placebo every two weeks at a 1:1 ratio. After 18 months, clinical deterioration in the Lecanemab group was 27% slower than in the placebo group, and the difference was significant. All secondary endpoints, including overall cognition, daily living abilities, quality of life, and caregiver burden, also reached statistically significant outcomes. Aβ-PET imaging indicated brain Aβ levels below the positive threshold, supporting a positive correlation between the extent of Aβ reduction and clinical benefit. In terms of biomarkers, except for neurofilament light chain (NfL), all cerebrospinal fluid and plasma biomarkers for Tau pathology and neurodegeneration were more favorable in the Lecanemab group, supporting the disease modification hypothesis [32]. Common adverse reactions during Lecanemab treatment included ARIA-E (with an overall incidence rate of 12.6%, of which symptomatic ARIA-E was 2.8%) and ARIA-H (with an incidence rate of 17.3%; symptomatic ARIA-H was at 0.7%) [33]. Two cases of potentially fatal cerebral hemorrhage associated with Lecanemab treatment, both accompanied by anticoagulation therapy, suggested that patients on anticoagulants might not be eligible for anti-Aβ monoclonal antibody treatment but further safety data to validate this result are pending [34]. In June 2023, the FDA approved Lecanemab for the treatment of mild cognitive impairment and mild dementia due to AD.

#### 2.1.3. Donanemab

Donanemab is a monoclonal antibody capable of binding to Aβ modified by pyroglutamate at the N-terminal second position, thereby facilitating rapid and complete clearance of amyloid deposits. The TRAILBLAZER-ALZ 2 (NCT04437511) study aimed to evaluate the efficacy and safety of Donanemab in early symptomatic AD patients, with participants stratified based on their levels of Tau protein, a predictive biomarker for AD progression. Among those with medium to low levels of Tau (n = 1182), the primary endpoint, measured by the integrated Alzheimer’s Disease Rating Scale (iADRS), showed that Donanemab slowed cognitive decline by 35% (*p* < 0.001). Additionally, the CDR-SB score showed that Donanemab slowed cognitive deterioration by 36% after 18 months [35,36]. Participants in the Donanemab group experienced a 40% reduction in the deterioration of daily living capabilities, and the risk of disease progression was decreased by 39%. After 18 months of treatment with Donanemab, there was an average reduction of 84% in Aβ plaques, compared to a 1% reduction in participants who received a placebo. In the Donanemab group, 24% of patients experienced ARIA-E, with 6% exhibiting symptoms; 31.4% of patients in the Donanemab group experienced ARIA-H. It is also noteworthy that in TRAILBLAZER-ALZ 2, there were three cases of death following severe ARIA events, and these were treatment-related. On 2 July 2024, Donanemab was approved by the FDA for the treatment of early symptomatic AD.

### 2.2. Aβ Monoclonal Antibodies in Clinical Trials

#### 2.2.1. Remternetug

As the successor of Donanemab, Remternetug represents the next generation of N3pG Aβ antibodies [37]. Results from the phase I clinical trials have shown that Remternetug can rapidly and steadily reduce amyloid plaques in AD patients. A dose-dependent reduction in Aβ was observed across all dosing groups, though the smaller 250 mg dose demonstrated a lesser response by day 85 compared to the higher doses (700–2800 mg). By day 169, Aβ clearance was achieved in 18 out of the 24 treated patients, and the phase III trial is expected to be completed in October 2025 (NCT04451408).

#### 2.2.2. SHR-1707

SHR-1707 is a novel humanized anti-Aβ IgG1 monoclonal antibody that binds to both Aβ fibrils and monomers, blocking the formation of Aβ plaques and promoting microglial phagocytosis of Aβ [38]. On 10 March 2021, the clinical trial application of SHR-1707, and anti-amyloid-β antibody, for the treatment of AD by Hengrui Medicine received tacit approval from the NMPA, making SHR-1707 the first domestically developed Aβ monoclonal antibody to be declared for clinical trials in China. The phase I study involving healthy adults demonstrated that SHR-1707 was safe and well-tolerated at doses ranging from 2 to 60 mg/kg [38]. Additionally, phase Ib (NCT06114745) and phase Ⅱ (CTR20234283, NCT06199037) clinical trials are further evaluating its efficacy in AD patients. Overall, SHR-1707 appears to be a promising candidate for AD treatment, especially given the favorable safety data obtained so far.

Despite some successes of the Aβ antibodies drugs, particularly the approved Lecanemab and Donanemab, these treatments still face several challenges. These include identifying the optimal timing for administration, managing dosages, ensuring long-term safety, and addressing the risks associated with ARIA [39]. We further discuss these important issues based on the currently limited evidence, emphasizing the urgent need for additional exploration and resolution in future clinical research.

### 2.3. Characteristics of Aβ Monoclonal Antibody Treatment

#### 2.3.1. Dosage of Treatment

It is widely recognized that only a small portion of the administered IgG antibodies reach the patient’s brain [40,41]. Therefore, the dosage of the antibody correlates well with the efficiency of Aβ clearance in the brain. However, high dosages of monoclonal antibodies are also associated with a higher incidence of ARIA. The method of administration can also have an impact on the efficacy. Compared to intravenous injections, subcutaneous injections may result in lower peak concentrations of circulating antibodies. To increase the efficiency of Aβ monoclonal antibodies crossing the blood–brain barrier (BBB), Rezai AR et al. from West Virginia University, Morgantown, WV, USA, explored the effects of ultrasound-induced BBB opening combined with Aducanumab monoclonal antibody treatment for AD [40]. The study involved the use of focused ultrasound to transiently open the BBB during the infusion of Aducanumab monoclonal antibody over six months, aiming to enhance Aβ removal in selected brain regions of three participants within this timeframe. The reduction in Aβ levels was numerically greater in the regions treated with focused ultrasound than in the contralateral hemisphere’s homologous areas not treated with focused ultrasound, as measured through [18F] fluorobetaben positron emission tomography scans.

#### 2.3.2. Timing of Treatment

The FDA-approved monoclonal antibodies Lecanemab and Donanemab were approved only for the treatment of AD-related MCI and mild AD, with better efficacy for AD-related MCI. However, for patients in the later stages of the disease, the effectiveness of monoclonal antibody treatment may be limited. Therefore, it is currently recommended to treat patients with AD-related MCI rather than waiting until they reach the dementia stage [31,42].

#### 2.3.3. Form of Aβ Clearance

Compared to Lecanemab monoclonal antibodies, which specifically target Aβ fibrils and protofibrils, Gantenerumab monoclonal antibodies primarily target Aβ deposits (i.e., plaques) and, to some extent, soluble Aβ monomers. The significance of differences in antibody specificity is not fully understood yet, but the first generation of monoclonal antibodies targeting Aβ monomers did not show significant clinical efficacy [26]. This supports the hypothesis that Aβ protofibrils represent a promising therapeutic target.

#### 2.3.4. Monitoring of ARIA

ARIA, observed in MRI, is transient in most cases during the treatment [43]. The biological mechanisms behind ARIA have not been fully elucidated but they are hypothesized to be due to increased rates of Aβ plaque clearance and vascular permeability, as well as the direct interaction between the monoclonal antibody and Aβ deposits on the vessel walls, which could cause damage. Clinical vigilance for ARIA should be at its highest following the commencement of monoclonal antibody treatment. Patients who show new symptoms suggestive of ARIA require routine monitoring with MRI and special scanning. Approximately 25% of ARIA cases are symptomatic [44]. For ARIA patients, a thorough evaluation with imaging and clinical presentations must be performed before re-administration.

## 3. New Drugs Targeting Tau Protein

Tau protein is a highly soluble, intrinsically disordered microtubule-associated protein, predominantly localized in axons, where it binds to microtubules, thereby stabilizing their structure. Due to its strong association with neurofibrillary tangle (NFT) pathology, neuronal hypometabolism, and degeneration in AD, Tau presents an attractive therapeutic target [45,46]. Various mechanisms of action have been proposed for treatment targeting Tau, including the specific removal of pathological Tau species, reducing the production of Tau protein, and promoting the physiological function of Tau by stabilizing microbubbles or inhibiting post-translational modifications. However, to date, no therapeutic strategy targeting Tau has demonstrated clear clinical efficacy in preclinical or early stages of AD patients [47,48].

### 3.1. Tau Aggregation Inhibition

HMTM (TRx0237) is a Tau aggregation inhibitor intended to reduce Tau pathology in patients with AD. The 24-month study data from the phase III clinical trial LUCIDITY (NCT03446001) revealed a significant reduction in disease progression in the participants compared to a control group [49]. Among early-stage disease subgroups, there was a significant decrease in patients progressing to the dementia stage of AD, indicating that HMTM has the potential to become the first oral anti-Tau protein therapy for treating AD. The LUCIDITY trial compared cognitive and functional outcomes, as well as changes in brain volume loss, within 12 months among participants with early to moderate AD who received a daily dose of 16 mg HMTM to those in a control group. An open-label phase was followed, where everyone received a daily treatment of 16 mg HMTM for the following 12 months. The blood biomarker data announced demonstrated that a daily dose of 16 mg HMTM reduced the change in levels of NfL in blood by 95% compared to the control group. Furthermore, early-stage participants treated with a daily dose of 16 mg HMTM showed cognitive indices significantly above baseline at 18 months, only returning to baseline values after 24 months. In this subgroup, the incidence of disease symptoms progressing to the dementia stage was significantly reduced compared to the control group. Moreover, further analysis indicated that despite the control group transitioning to receive 12 months of 16 mg HMTM treatment during the open-label phase, the assessed indices were still significantly below the baseline (based on the ADAS-Cog13 scale assessment).

### 3.2. Reducing the Expression of Tau Protein

Antisense oligonucleotides (ASOs) were used to target and degrade the Tau mRNA transcription template (MAPTRx, BIIB080). In preclinical mouse models, ASOs targeting Tau reduced Tau protein levels and reversed pathological Tau protein deposition. The above phase Ib clinical trial demonstrated the safety of targeting Tau protein with ASOs in humans, with total Tau and pTau181 showing a dose-dependent decrease. Another study is underway to determine whether targeting Tau with ASOs can slow down the rate of cognitive decline in patients with mild cognitive impairment or mild AD (NCT05399888) now [50].

### 3.3. Tau Immunotherapy

Active and passive immunization strategies targeting pathogenic forms of Tau protein are currently under development. Active immunotherapy employs Tau immunogens as a vaccine [51]. However, since the Tau protein is an endogenous protein, there may be potential adverse autoimmune reactions. AADvac1, an active vaccine designed to target the truncated N-terminus of the Tau fragment, have demonstrated good immunogenicity and safety profiles [52]. ACI-35 is another vaccine targeting the Tau protein, and its second-generation product ACI-35.030 could specifically target phosphorylated Tau protein and exhibit enhanced immunogenicity compared to its first-generation counterpart [53]. Passive immunotherapy targets specific antigen epitopes, and the responses usually subside after antibody clearance, making passive immunity reversible, which may increase the risk of unique phenotype reactions and associated adverse effects. Currently, some antibodies are being utilized in passive immunotherapy. JNJ-63733657 is an IgG1 antibody with high affinity for p-Tau 217 capable of neutralizing Tau seeds and inhibiting the spread of Tau pathology [54,55]. Bepranemab (UCB0107) is a humanized monoclonal IgG4 antibody that specifically binds to the amino acids 235–250 near the microtubule-binding region of Tau and blocks Tau production. TauE2814 is a humanized monoclonal IgG1 antibody that specifically recognizes the HVPGG epitope within the microtubule-binding region of the Tau protein [56]. It binds to extracellular Tau and reduces levels of free Tau containing the central domain. However, it remains unclear whether passive immunization with anti-Tau antibodies in patients can only target extracellular pathological Tau proteins or if these antibodies can reach the cytoplasm of neurons without modification. Additionally, a recent analysis indicated that local production of Tau protein (rather than spreading between brain regions) drives the accumulation of Tau deposition and the formation of neurofibrillary tangles as cognitive impairment progresses [57]. The efficacy of current forms of anti-Tau antibody treatments remains uncertain since it has not been established whether the pathological forms of extracellular Tau proteins drive the spread of Tau pathology.

Overall, tau-targeted treatments, focusing on tau tangles associated with neuronal dysfunction, are still in early development, with their efficacy not yet fully validated [58]. Both strategies still face some challenges, such as patient heterogeneity and disease stage variability [59,60]. Thus, despite their potential, amyloid and tau-targeted therapies need further refinement to achieve consistent, clinically meaningful outcomes for AD patients.

## 4. New Drugs: Beyond the Amyloid and Tau Proteins

### 4.1. Inflammation

Candidates with therapeutic approaches aim at reducing neuroinflammation constitute the largest part of the AD drug development pipelines, second only to neurotransmitter modulators and Aβ-targeted drugs. While most of these drugs possess anti-inflammatory characteristics, a minority activate immune responses to enhance the clearance of aggregates are involved too [61].

#### 4.1.1. NE-3107

The central mechanism of NE-3107 involves protecting neurons by reducing inflammation and enhancing glucose utilization, ultimately improving brain health. Specifically, NE-3107 selectively inhibits the inflammatory ERK signaling pathway, reducing neuroinflammation by targeting inflammation-driven insulin resistance and primary pathological inflammatory cascades. NE-3107 is currently undergoing a phase III trial for AD (NCT04669028) [62]. Previous phase II clinical trial results have shown improvements in cognitive functions and biomarker levels in AD patients following treatment with NE-3107.

#### 4.1.2. Masitinib

Masitinib is an oral tyrosine kinase inhibitor. It may remodel the neuronal microenvironment, transitioning the neuroimmune system from a neurotoxic state to a neuroprotective state and exhibiting neuroprotective effects in neurodegenerative diseases by inhibiting the activity of mast cells and microglia/macrophages [63]. Masitinib inhibits the activation of mast cells by specifically inhibiting the activity of the protein kinases c-Kit, Lyn, and Fyn. Additionally, it targets microglia/macrophages by inhibiting the type 1 macrophage colony-stimulating factor receptor (MCSFR-1) [64]. In a phase III clinical trial involving patients with mild to moderate AD, masitinib demonstrated significant slowing of cognitive decline compared to placebo. This provided the first clinical evidence that targeting innate immune cells and represented a possible effective treatment approach for later-stage dementia caused by AD [65].

#### 4.1.3. Simufilam

Simufilam is an orally administered small molecule compound that targets filamin A (FLNA), a scaffolding protein highly expressed in the brain [66]. In the brains of AD patients, FLNA undergoes a conformational change, enabling Aβ42 peptide fragments to bind to receptors, leading to the production of hyperphosphorylated Tau protein and the release of inflammatory cytokines. By binding to FLNA and restoring its normal conformation and function, Simufilam interrupts the signaling initiated by Aβ42, thereby holding the potential to reduce both neurodegeneration and neuroinflammation by targeting a single point [67]. Positive results were achieved in phase II clinical trials for Simufilam; not only did it show good safety and tolerability but also, in terms of exploratory efficacy endpoints, 47% of treated patients demonstrated a 4.7-point improvement in ADAS-Cog scores one year after treatment compared to baseline. Simufilam is currently undergoing phase III clinical trials (NCT05575076) to evaluate its effectiveness in treating patients with mild to moderate AD.

### 4.2. Synaptic Plasticity/Neuroprotection

Seeking drugs that enhance synaptic plasticity or confer neuroprotection is among the most common candidate therapeutics in the AD drug development pipelines. A unique aspect of clinical trials for synaptic drugs is that enhanced synaptic function could render disease modification or might improve cognitive or behavioral outcomes, yielding symptomatic benefits.

#### 4.2.1. AR1001

AR1001 is a small-molecule inhibitor targeting phosphodiesterase-5 (PDE-5), and it has been proven to exert neuroprotective effects by inhibiting neuronal apoptosis and restoring synaptic plasticity [68]. A phase 2 study of AR1001 in patients with mild to moderate AD showed that after 52 weeks of treatment, Alzheimer’s Disease Cooperative Study–Clinical Global Impression of Change (ADCS-CGIC) scores decreased by 1.17 points (10 mg group) and 0.76 points (30 mg group) compared to baseline, respectively. For mild AD patients treated solely with AR1001, participants in the 10 mg group showed a 2.4-point (15.1%) improvement in scores, while participants in the 30 mg group demonstrated an 8.7-point improvement (46.3%, *p* = 0.001) compared to baseline at 52 weeks. Following the confirmation of AR1001′s therapeutic benefits in patients with mild AD(NCT03625622), a phase 3 clinical trial is being conducted in early-stage AD populations to test its safety and efficacy.

#### 4.2.2. Sigma-1 Receptor (S1R)

S1R is involved in all major neurotransmitter systems and changes in its function and/or expression have been associated with AD and other neurodegenerative diseases [69]. S1Rs are recently explored targets for AD since at the early stages of AD, there is a notable deficiency in the expression of S1R in the brain. In recent years, there has been a significant amount of preclinical and clinical research on S1R and sigma-2 receptor (S2R) ligands for the treatment of neurological diseases. Studies have revealed that therapeutic effects are mediated through the regulation of S1R, thereby impacting oxidative stress, Tau protein phosphorylation, autophagy, and calcium homeostasis [70,71,72,73]. Investigational S1R/S2R drugs such as Blarcamesine (ANAVEX2-73), ANAVEX3-71, and CT1812 have shown promising results in clinical trials. Blarcamesine (ANAVEX2-73) is an orally available small-molecule agonist targeting S1R which can effectively improve mitochondrial activation levels within neuronal cells and ameliorate neuroinflammation. ANAVEX2-73 has completed phase IIa and phase IIb/III clinical trials for AD. In a phase IIb/III trial involving 509 patients, ANAVEX2-73 met its primary endpoints and key secondary endpoints (NCT03790709). Compared to the placebo group, the treatment group showed a reduction of 1.85 in ADAS-Cog score (*p* = 0.033) and a 0.42 decrease in Clinical Dementia Rating Scale (CDR-SB) score (*p* = 0.040) in patients with mild cognitive impairment. In terms of safety, the most common adverse effect of ANAVEX2-73 was dizziness. The open-label extension study ATTENTION-AD will further assess the efficacy and safety of ANAVEX2-73 in treatments with a period of lasting over 96 weeks (NCT04314934) [74]. ANAVEX3-71 (AF710B) is an agonist for both the muscarinic M1 receptor and S1R. Preclinical studies indicated that ANAVEX3-71 could alter disease characteristics such as cognitive deficits and amyloid and Tau pathology in AD transgenic mice (3xTg-AD), exert beneficial effects on mitochondrial dysfunction, and attenuate neuroinflammation. In a reported phase I clinical trial (NCT04442945), oral administration of 5–200 mg per day did not result in any severe adverse events. The pharmacokinetics (serum AF710B concentrations) of ANAVEX3-71 also correlated well and linearly with doses up to 160 mg [75]. CT1812 is an orally active investigational small molecule that can cross the blood–brain barrier and selectively binds to the S2R complex. It prevents Aβ oligomers from binding to neurons and promotes their dissociation from the neuronal receptors by interacting with S2R without affecting Aβ monomer levels. In phase I clinical trials, minimal adverse events were observed in healthy volunteers at doses up to 560 mg, indicating the relative safety profile of the compounds (NCT03716427 and NCT02907567) [76]. In a placebo-controlled biomarker study involving patients with mild AD, the concentration of Aβ oligomers in the cerebrospinal fluid significantly increased in the CT1812 treatment group compared to those receiving a placebo, whereas the synaptic protein fragments neurogranin and synaptosomal-associated protein were reduced relative to baseline [77]. CT1812 is currently undergoing the phase II START study (NCT05531656), which aims to evaluate the therapeutic effects and drug tolerance of CT1812 in AD patients.

## 5. Conclusions

In conclusion, with the introduction of Aβ-targeting therapies and other monoclonal antibodies, the treatment of AD is entering a transformative phase. Advancements in non-Aβ/Tau pathways and ongoing clinical trials also offer promising improvements in patient outcomes, with continued research critical for future progress.

## Figures and Tables

**Table 1:** New drugs for Alzheimer’s disease.

Mechanism of Action	Drug Name	Targets of Drug Action	Effectiveness	Safety	Research Enterprise	Present Stage
Aβ Monoclonal Antibodies	Aducanumab	oligomers, fibrils, plaques	yes	low	Biogen/Eisai	Approvable for marketing (2021)
	Lecanemab	oligomers, fibrils, plaques	yes	high	Biogen/Eisai	Full approval for marketing (2023)
	Donanemab	plaques	yes	moderate	Eli Lilly	Full approval for marketing (2024)
	Remternetug	N3pG Aβ	yes	moderate	Eli Lilly	Phase III
	SHR-1707	Aβ monomer	yes	/	Hengrui	Phase III
Targeting Tau Protein	HMTM (TRx0237)	tau, TDP-43, and synuclein	uncertain	uncertain	Taurx Therapeutics	Phase III
	MAPTRx	antisense oligonucleotide	uncertain	uncertain	Biogen	Phase II
	ACI-35.030	p-tau	uncertain	uncertain	AC Immune	Phase II
	AADvac-1	tau	uncertain	uncertain	Axon Neuroscience SE	Phase II
	JNJ-63733657	tau	uncertain	uncertain	Janssen	Phase II
	Bepranemab	tau	uncertain	uncertain	Hoffmann-La Roche	Phase II
	E2814	tau	uncertain	uncertain	Eisai Co	Phase I b/IIa
Inflammation	NE3107	anti-inflammatory	uncertain	uncertain	BioVie Pharma	Phase III
	Masitinib	innate immune cells	uncertain	uncertain	AB Science	Phase III
	Simufilam	filamin A	uncertain	uncertain	Cassava Sciences	Phase III
Synaptic Plasticity/Neuroprotection	Anavex 2-73	M1 and σ1 receptor	uncertain	uncertain	Anavex Life Science Corp	Phase IIb/III
	Anavex 3-71	M1 receptor	uncertain	uncertain	Anavex Life Sciences Corp	Phase II
	CT1812	Sigma-2 Receptor	uncertain	uncertain	Cognition Therapeutics Inc	Phase II
	AR1001	Phosphodiesterase-5	uncertain	uncertain	Aribio Co	Phase III
Exosomes	ahaMSC-Exos	exosomes	uncertain	uncertain	Shanghai Jiaotong University	Phase I b/IIa
Psychiatric Abnormalities Associated with AD	Rexulti	agitation	yes	high	Otsuka Pharmaceutical/Lundbeck	Full approval for marketing (2023)
	KarXT	hallucinations and delusions	yes	high	Karuna Therapeutics	Phase III

Aβ: Amyloid Beta; N3pG Aβ: Aβ with N-terminal Asp3 pyroglutamation; M1 receptor: Muscarinic Acetylcholine Receptor Type 1; p-tau: phosphorylated tau.

## Data Availability

No new data were created or analyzed in this study.

## References

[1] Selkoe D.J., Hardy J. (2016). The amyloid hypothesis of Alzheimer’s disease at 25 years. EMBO Mol. Med..

[2] Jucker M., Walker L.C. (2011). Pathogenic protein seeding in Alzheimer disease and other neurodegenerative disorders. Ann. Neurol..

[3] Birks J.S., Harvey R.J. (2018). Donepezil for dementia due to Alzheimer’s disease. Cochrane Database Syst. Rev..

[4] Marucci G., Buccioni M., Ben D.D., Lambertucci C., Volpini R., Amenta F. (2021). Efficacy of acetylcholinesterase inhibitors in Alzheimer’s disease. Neuropharmacology.

[5] Birks J., Grimley Evans J., Iakovidou V., Tsolaki M., Holt F.E. (2009). Rivastigmine for Alzheimer’s disease. Cochrane Database Syst. Rev..

[6] Reisberg B., Doody R., Stöffler A., Schmitt F., Ferris S., Möbius H.J. (2003). Memantine in moderate-to-severe Alzheimer’s disease. N. Engl. J. Med..

[7] Zou B., Li J., Ma R.X., Cheng X.Y., Ma R.Y., Zhou T.Y., Wu Z.Q., Yao Y., Li J. (2023). Gut Microbiota is an Impact Factor based on the Brain-Gut Axis to Alzheimer’s Disease: A Systematic Review. Aging Dis..

[8] Xiao G., Tang R., Yang N., Chen Y. (2023). Review on pharmacological effects of gastrodin. Arch. Pharmacal. Res..

[9] Cai Y., Liu J., Wang B., Sun M., Yang H. (2022). Microglia in the Neuroinflammatory Pathogenesis of Alzheimer’s Disease and Related Therapeutic Targets. Front. Immunol..

[10] Zhao Z., Liu Y., Ruan S., Hu Y. (2023). Current Anti-Amyloid-β Therapy for Alzheimer’s Disease Treatment: From Clinical Research to Nanomedicine. Int. J. Nanomed..

[11] Briggs R., Kennelly S.P., O’Neill D. (2016). Drug treatments in Alzheimer’s disease. Clin. Med..

[12] Mangialasche F., Solomon A., Winblad B., Mecocci P., Kivipelto M. (2010). Alzheimer’s disease: Clinical trials and drug development. Lancet Neurol..

[13] Tabor G.T., Holtzman D.M. (2023). Current status of amyloid-targeting immunotherapies for Alzheimer’s disease. Sci. Transl. Med..

[14] Barakos J., Purcell D., Suhy J., Chalkias S., Burkett P., Marsica Grassi C., Castrillo-Viguera C., Rubino I., Vijverberg E. (2022). Detection and Management of Amyloid-Related Imaging Abnormalities in Patients with Alzheimer’s Disease Treated with Anti-Amyloid Beta Therapy. J. Prev. Alzheimer’s Dis..

[15] Mohamed T., Shakeri A., Rao P.P. (2016). Amyloid cascade in Alzheimer’s disease: Recent advances in medicinal chemistry. Eur. J. Med. Chem..

[16] Xia W., Wong S.T., Hanlon E., Morin P. (2012). γ-Secretase modulator in Alzheimer’s disease: Shifting the end. J. Alzheimer’s Dis..

[17] Coric V., van Dyck C.H., Salloway S., Andreasen N., Brody M., Richter R.W., Soininen H., Thein S., Shiovitz T., Pilcher G. (2012). Safety and tolerability of the γ-secretase inhibitor avagacestat in a phase 2 study of mild to moderate Alzheimer disease. Arch. Neurol..

[18] Walker J.R., Pacoma R., Watson J., Ou W., Alves J., Mason D.E., Peters E.C., Urbina H.D., Welzel G., Althage A. (2013). Enhanced proteolytic clearance of plasma Aβ by peripherally administered neprilysin does not result in reduced levels of brain Aβ in mice. J. Neurosci..

[19] Qian C., Yang C., Lu M., Bao J., Shen H., Deng B., Li S., Li W., Zhang M., Cao C. (2021). Activating AhR alleviates cognitive deficits of Alzheimer’s disease model mice by upregulating endogenous Aβ catabolic enzyme Neprilysin. Theranostics.

[20] Jucker M., Walker L.C. (2023). Alzheimer’s disease: From immunotherapy to immunoprevention. Cell.

[21] Song C., Shi J., Zhang P., Zhang Y., Xu J., Zhao L., Zhang R., Wang H., Chen H. (2022). Immunotherapy for Alzheimer’s disease: Targeting β-amyloid and beyond. Transl. Neurodegener..

[22] Salloway S., Sperling R., Fox N.C., Blennow K., Klunk W., Raskind M., Sabbagh M., Honig L.S., Porsteinsson A.P., Ferris S. (2014). Two phase 3 trials of bapineuzumab in mild-to-moderate Alzheimer’s disease. N. Engl. J. Med..

[23] Bateman R.J., Smith J., Donohue M.C., Delmar P., Abbas R., Salloway S., Wojtowicz J., Blennow K., Bittner T., Black S.E. (2023). Two Phase 3 Trials of Gantenerumab in Early Alzheimer’s Disease. N. Engl. J. Med..

[24] Sperling R.A., Donohue M.C., Raman R., Rafii M.S., Johnson K., Masters C.L., van Dyck C.H., Iwatsubo T., Marshall G.A., Yaari R. (2023). Trial of Solanezumab in Preclinical Alzheimer’s Disease. N. Engl. J. Med..

[25] Ostrowitzki S., Bittner T., Sink K.M., Mackey H., Rabe C., Honig L.S., Cassetta E., Woodward M., Boada M., van Dyck C.H. (2022). Evaluating the Safety and Efficacy of Crenezumab vs Placebo in Adults With Early Alzheimer Disease: Two Phase 3 Randomized Placebo-Controlled Trials. JAMA Neurol..

[26] Söderberg L., Johannesson M., Nygren P., Laudon H., Eriksson F., Osswald G., Möller C., Lannfelt L. (2023). Lecanemab, Aducanumab, and Gantenerumab—Binding Profiles to Different Forms of Amyloid-Beta Might Explain Efficacy and Side Effects in Clinical Trials for Alzheimer’s Disease. Neurotherapeutics.

[27] Sevigny J., Chiao P., Bussière T., Weinreb P.H., Williams L., Maier M., Dunstan R., Salloway S., Chen T., Ling Y. (2016). The antibody aducanumab reduces Aβ plaques in Alzheimer’s disease. Nature.

[28] Salloway S., Chalkias S., Barkhof F., Burkett P., Barakos J., Purcell D., Suhy J., Forrestal F., Tian Y., Umans K. (2022). Amyloid-Related Imaging Abnormalities in 2 Phase 3 Studies Evaluating Aducanumab in Patients with Early Alzheimer Disease. JAMA Neurol..

[29] Budd Haeberlein S., Aisen P.S., Barkhof F., Chalkias S., Chen T., Cohen S., Dent G., Hansson O., Harrison K., von Hehn C. (2022). Two Randomized Phase 3 Studies of Aducanumab in Early Alzheimer’s Disease. J. Prev. Alzheimer’s Dis..

[30] van Dyck C.H., Swanson C.J., Aisen P., Bateman R.J., Chen C., Gee M., Kanekiyo M., Li D., Reyderman L., Cohen S. (2023). Lecanemab in Early Alzheimer’s Disease. N. Engl. J. Med..

[31] Cohen S., van Dyck C.H., Gee M., Doherty T., Kanekiyo M., Dhadda S., Li D., Hersch S., Irizarry M., Kramer L.D. (2023). Lecanemab Clarity AD: Quality-of-Life Results from a Randomized, Double-Blind Phase 3 Trial in Early Alzheimer’s Disease. J. Prev. Alzheimer’s Dis..

[32] Swanson C.J., Zhang Y., Dhadda S., Wang J., Kaplow J., Lai R.Y.K., Lannfelt L., Bradley H., Rabe M., Koyama A. (2021). A randomized, double-blind, phase 2b proof-of-concept clinical trial in early Alzheimer’s disease with lecanemab, an anti-Aβ protofibril antibody. Alzheimer’s Res. Ther..

[33] McDade E., Cummings J.L., Dhadda S., Swanson C.J., Reyderman L., Kanekiyo M., Koyama A., Irizarry M., Kramer L.D., Bateman R.J. (2022). Lecanemab in patients with early Alzheimer’s disease: Detailed results on biomarker, cognitive, and clinical effects from the randomized and open-label extension of the phase 2 proof-of-concept study. Alzheimer’s Res. Ther..

[34] Pfeifer M., Boncristiano S., Bondolfi L., Stalder A., Deller T., Staufenbiel M., Mathews P.M., Jucker M. (2002). Cerebral hemorrhage after passive anti-Abeta immunotherapy. Science.

[35] Mintun M.A., Lo A.C., Duggan Evans C., Wessels A.M., Ardayfio P.A., Andersen S.W., Shcherbinin S., Sparks J., Sims J.R., Brys M. (2021). Donanemab in Early Alzheimer’s Disease. N. Engl. J. Med..

[36] Sims J.R., Zimmer J.A., Evans C.D., Lu M., Ardayfio P., Sparks J., Wessels A.M., Shcherbinin S., Wang H., Monkul Nery E.S. (2023). Donanemab in Early Symptomatic Alzheimer Disease: The TRAILBLAZER-ALZ 2 Randomized Clinical Trial. JAMA.

[37] Maheshwari S., Singh A., Ansari V.A., Mahmood T., Wasim R., Akhtar J., Verma A. (2024). Navigating the dementia landscape: Biomarkers and emerging therapies. Ageing Res. Rev..

[38] Hu W., Shakib S., Williams J., Fan Y., Zhang Q., Qin H., Wu J., Zhang X., Liu Y., Zhou R. (2023). Safety, tolerability, pharmacokinetics and pharmacodynamics of a single intravenous dose of SHR-1707 in healthy adult subjects: Two randomized, double-blind, phase 1 studies. J. Alzheimer’s Dis..

[39] Cummings J., Osse A.M.L., Cammann D., Powell J., Chen J. (2023). Anti-Amyloid Monoclonal Antibodies for the Treatment of Alzheimer’s Disease. BioDrugs.

[40] Rezai A.R., D’Haese P.F., Finomore V., Carpenter J., Ranjan M., Wilhelmsen K., Mehta R.I., Wang P., Najib U., Vieira Ligo Teixeira C. (2024). Ultrasound Blood-Brain Barrier Opening and Aducanumab in Alzheimer’s Disease. N. Engl. J. Med..

[41] Pardridge W.M. (2020). Treatment of Alzheimer’s Disease and Blood-Brain Barrier Drug Delivery. Pharmaceuticals.

[42] Shcherbinin S., Evans C.D., Lu M., Andersen S.W., Pontecorvo M.J., Willis B.A., Gueorguieva I., Hauck P.M., Brooks D.A., Mintun M.A. (2022). Association of Amyloid Reduction After Donanemab Treatment with Tau Pathology and Clinical Outcomes: The TRAILBLAZER-ALZ Randomized Clinical Trial. JAMA Neurol..

[43] Roytman M., Mashriqi F., Al-Tawil K., Schulz P.E., Zaharchuk G., Benzinger T.L.S., Franceschi A.M. (2023). Amyloid-Related Imaging Abnormalities: An Update. AJR Am. J. Roentgenol..

[44] Barakos J., Sperling R., Salloway S., Jack C., Gass A., Fiebach J.B., Tampieri D., Melançon D., Miaux Y., Rippon G. (2013). MR imaging features of amyloid-related imaging abnormalities. AJNR Am. J. Neuroradiol..

[45] Jouanne M., Rault S., Voisin-Chiret A.S. (2017). Tau protein aggregation in Alzheimer’s disease: An attractive target for the development of novel therapeutic agents. Eur. J. Med. Chem..

[46] Mummery C.J., Börjesson-Hanson A., Blackburn D.J., Vijverberg E.G.B., De Deyn P.P., Ducharme S., Jonsson M., Schneider A., Rinne J.O., Ludolph A.C. (2023). Tau-targeting antisense oligonucleotide MAPT(Rx) in mild Alzheimer’s disease: A phase 1b, randomized, placebo-controlled trial. Nat. Med..

[47] Sinsky J., Pichlerova K., Hanes J. (2021). Tau Protein Interaction Partners and Their Roles in Alzheimer’s Disease and Other Tauopathies. Int. J. Mol. Sci..

[48] Avila J., Lucas J.J., Perez M., Hernandez F. (2004). Role of tau protein in both physiological and pathological conditions. Physiol. Rev..

[49] Wischik C.M., Bentham P., Gauthier S., Miller S., Kook K., Schelter B.O. (2022). Oral Tau Aggregation Inhibitor for Alzheimer’s Disease: Design, Progress and Basis for Selection of the 16 mg/day Dose in a Phase 3, Randomized, Placebo-Controlled Trial of Hydromethylthionine Mesylate. J. Prev. Alzheimer’s Dis..

[50] Self W.K., Holtzman D.M. (2023). Emerging diagnostics and therapeutics for Alzheimer disease. Nat. Med..

[51] Wiser I., Balicer R.D., Cohen D. (2007). An update on smallpox vaccine candidates and their role in bioterrorism related vaccination strategies. Vaccine.

[52] Novak P., Kovacech B., Katina S., Schmidt R., Scheltens P., Kontsekova E., Ropele S., Fialova L., Kramberger M., Paulenka-Ivanovova N. (2021). ADAMANT: A placebo-controlled randomized phase 2 study of AADvac1, an active immunotherapy against pathological tau in Alzheimer’s disease. Nat. Aging.

[53] Godyń J., Jończyk J., Panek D., Malawska B. (2016). Therapeutic strategies for Alzheimer’s disease in clinical trials. Pharmacol. Rep..

[54] Panza F., Dibello V., Sardone R., Castellana F., Zupo R., Lampignano L., Bortone I., Stallone R., Cirillo N., Damiani C. (2023). Clinical development of passive tau-based immunotherapeutics for treating primary and secondary tauopathies. Expert Opin. Investig. Drugs.

[55] Congdon E.E., Ji C., Tetlow A.M., Jiang Y., Sigurdsson E.M. (2023). Tau-targeting therapies for Alzheimer disease: Current status and future directions. Nat. Rev. Neurol..

[56] Roberts M., Sevastou I., Imaizumi Y., Mistry K., Talma S., Dey M., Gartlon J., Ochiai H., Zhou Z., Akasofu S. (2020). Pre-clinical characterisation of E2814, a high-affinity antibody targeting the microtubule-binding repeat domain of tau for passive immunotherapy in Alzheimer’s disease. Acta Neuropathol. Commun..

[57] Lövestam S., Li D., Wagstaff J.L., Kotecha A., Kimanius D., McLaughlin S.H., Murzin A.G., Freund S.M.V., Goedert M., Scheres S.H.W. (2024). Disease-specific tau filaments assemble via polymorphic intermediates. Nature.

[58] Wang Y., Mandelkow E. (2016). Tau in physiology and pathology. Nat. Rev. Neurosci..

[59] The L. (2022). Lecanemab for Alzheimer’s disease: Tempering hype and hope. Lancet.

[60] Mead S., Fox N.C. (2023). Lecanemab slows Alzheimer’s disease: Hope and challenges. Lancet Neurol..

[61] Twarowski B., Herbet M. (2023). Inflammatory Processes in Alzheimer’s Disease-Pathomechanism, Diagnosis and Treatment: A Review. Int. J. Mol. Sci..

[62] Reading C.L., Ahlem C.N., Murphy M.F. (2021). NM101 Phase III study of NE3107 in Alzheimer’s disease: Rationale, design and therapeutic modulation of neuroinflammation and insulin resistance. Neurodegener. Dis. Manag..

[63] McShane R., Westby M.J., Roberts E., Minakaran N., Schneider L., Farrimond L.E., Maayan N., Ware J., Debarros J. (2019). Memantine for dementia. Cochrane Database Syst. Rev..

[64] Ettcheto M., Cano A., Sanchez-López E., Verdaguer E., Folch J., Auladell C., Camins A. (2021). Masitinib for the treatment of Alzheimer’s disease. Neurodegener. Dis. Manag..

[65] Dubois B., López-Arrieta J., Lipschitz S., Doskas T., Spiru L., Moroz S., Venger O., Vermersch P., Moussy A., Mansfield C.D. (2023). Masitinib for mild-to-moderate Alzheimer’s disease: Results from a randomized, placebo-controlled, phase 3, clinical trial. Alzheimer’s Res. Ther..

[66] Wang H.Y., Cecon E., Dam J., Pei Z., Jockers R., Burns L.H. (2023). Simufilam Reverses Aberrant Receptor Interactions of Filamin A in Alzheimer’s Disease. Int. J. Mol. Sci..

[67] Wang H.Y., Pei Z., Lee K.C., Nikolov B., Doehner T., Puente J., Friedmann N., Burns L.H. (2023). Simufilam suppresses overactive mTOR and restores its sensitivity to insulin in Alzheimer’s disease patient lymphocytes. Front. Aging.

[68] Kang B.W., Kumar A., Song D.-K., Ha J.-Y., Choung J.J. (2023). Protective Effects of AR1001 in Alzheimer’s Disease Models: Polypharmacological Mechanisms. J. Alzheimer’s Dis..

[69] Ryskamp D.A., Korban S., Zhemkov V., Kraskovskaya N., Bezprozvanny I. (2019). Neuronal Sigma-1 Receptors: Signaling Functions and Protective Roles in Neurodegenerative Diseases. Front. Neurosci..

[70] Tsai S.Y., Pokrass M.J., Klauer N.R., Nohara H., Su T.P. (2015). Sigma-1 receptor regulates Tau phosphorylation and axon extension by shaping p35 turnover via myristic acid. Proc. Natl. Acad. Sci. USA.

[71] Christ M.G., Huesmann H., Nagel H., Kern A., Behl C. (2019). Sigma-1 Receptor Activation Induces Autophagy and Increases Proteostasis Capacity In Vitro and In Vivo. Cells.

[72] Delprat B., Crouzier L., Su T.P., Maurice T. (2020). At the Crossing of ER Stress and MAMs: A Key Role of Sigma-1 Receptor?. Adv. Exp. Med. Biol..

[73] Pannuzzo M. (2016). On the physiological/pathological link between Aβ peptide, cholesterol, calcium ions and membrane deformation: A molecular dynamics study. Biochim. Biophys. Acta.

[74] Hampel H., Caraci F., Cuello A.C., Caruso G., Nisticò R., Corbo M., Baldacci F., Toschi N., Garaci F., Chiesa P.A. (2020). A Path Toward Precision Medicine for Neuroinflammatory Mechanisms in Alzheimer’s Disease. Front. Immunol..

[75] Orciani C., Do Carmo S., Foret M.K., Hall H., Bonomo Q., Lavagna A., Huang C., Cuello A.C. (2023). Early treatment with an M1 and sigma-1 receptor agonist prevents cognitive decline in a transgenic rat model displaying Alzheimer-like amyloid pathology. Neurobiol. Aging.

[76] Grundman M., Morgan R., Lickliter J.D., Schneider L.S., DeKosky S., Izzo N.J., Guttendorf R., Higgin M., Pribyl J., Mozzoni K. (2019). A phase 1 clinical trial of the sigma-2 receptor complex allosteric antagonist CT1812, a novel therapeutic candidate for Alzheimer’s disease. Alzheimer’s Dement..

[77] LaBarbera K.M., Sheline Y.I., Izzo N.J., Yuede C.M., Waybright L., Yurko R., Edwards H.M., Gardiner W.D., Blennow K., Zetterberg H. (2023). A phase 1b randomized clinical trial of CT1812 to measure Aβ oligomer displacement in Alzheimer’s disease using an indwelling CSF catheter. Transl. Neurodegener..

